# The ‘One in a Million’ study: creating a database of UK primary care consultations

**DOI:** 10.3399/bjgp17X690521

**Published:** 2017-04-11

**Authors:** Marcus Jepson, Chris Salisbury, Matthew J Ridd, Chris Metcalfe, Ludivine Garside, Rebecca K Barnes

**Affiliations:** Centre for Academic Primary Care, School of Social and Community Medicine, University of Bristol, Bristol.; Centre for Academic Primary Care, School of Social and Community Medicine, University of Bristol, Bristol.; Centre for Academic Primary Care, School of Social and Community Medicine, University of Bristol, Bristol.; Centre for Academic Primary Care, School of Social and Community Medicine, University of Bristol, Bristol.; Centre for Academic Primary Care, School of Social and Community Medicine, University of Bristol, Bristol.; Centre for Academic Primary Care, School of Social and Community Medicine, University of Bristol, Bristol.

**Keywords:** data sharing, databases, factual, general practice, office visits, physician–patient relations, physicians, primary health care

## Abstract

**Background:**

Around 1 million primary care consultations happen in England every day. Despite this, much of what happens in these visits remains a ‘black box’.

**Aim:**

To create an archive of videotaped consultations and linked data based on a large sample of routine face-to-face doctor–patient consultations with consent for use in future research and training.

**Design and setting:**

Cross-sectional study in 12 general practices in the west of England, UK.

**Method:**

Up to two GPs from each practice took part in the study. Over 1 to 2 days, consecutive patients were approached until up to 20 eligible patients for each GP consented to be videotaped. Eligible patients were aged ≥18 years, consulting on their own behalf, fluent in English, and with capacity to consent. GP questionnaires were self-administered. Patient questionnaires were self-administered immediately pre-consultation and post-consultation, and GPs filled in a checklist after each recording. A follow-up questionnaire was sent to patients after 10 days, and data about subsequent related consultations were collected from medical records 3 months later.

**Results:**

Of the 485 patients approached, 421 (86.8%) were eligible. Of the eligible patients, 334 (79.3%) consented to participate and 327 consultations with 23 GPs were successfully taped (307 video, 20 audio-only). Most patients (*n* = 300, 89.8%) consented to use by other researchers, subject to specific ethical approval.

**Conclusion:**

Most patients were willing to allow their consultations to be videotaped, and, with very few exceptions, to allow recordings and linked data to be stored in a data repository for future use for research and training.

## INTRODUCTION

General practice accounts for over 85% of all NHS doctor–patient encounters, and in England alone 37 000 GPs^1^ see around 1 million patients every day across 7800 diverse practice settings.^2^ Despite an increase in telephone consulting, 90% of patient consultations still happen face-to-face in GP surgeries.^3^

Understanding of what happens in these encounters is limited because of a relative lack of research in situ. This is often attributed to perceived difficulties in recording routine consultations, including obtaining ethical permissions, recruiting participants and organising data collection, safe transfer, and storage. Most recent consultations research (2003) has been based on patient interviews, surveys, or medical records, which provide a limited and potentially biased account of what actually occurs.^4^ Consequently, the skill-sets necessary for working with consultations data have had fewer opportunities to be passed on and may be in danger of declining.

In contrast, some of the earliest and most influential studies in the history of general practice research were based on the study of directly observed or recorded consultations. One such study by Byrne and Long,^5^ published in 1976 but still widely referenced today, remains remarkable for several reasons: for demonstrating the acceptability and feasibility of collecting large datasets of recordings of ‘live’ consultations between doctors and patients in the UK; as the first real evidence base capturing the difference between doctor-centred and patient-centred care; and for capturing a snapshot of what is now our primary care heritage. Despite early critiques of their analytic approach,^6^ Byrne and Long remains a groundbreaking study. Furthermore, UK training for general practice has been heavily influenced by the use of videotaped consultations.^7^

Recently, there has been a rise of interest and investment in large datasets of primary health care, ‘Big-data’; that is, indirect records of selected information collected around the consultation. For example, the Clinical Practice Research Datalink^8^ contains anonymised medical records data for over 11 million patients. By contrast, no UK datasets currently exist of primary care consultations themselves that might enable capacity building (in terms of knowledge and skills) to improve consultation outcomes. Although other researchers have collected recordings of UK primary care consultations, most of these have relied on audio-only and have not been collected with data sharing in mind. As such, the data have been subject to restricted ethical permissions.^9^

The acceptability and feasibility of collecting audiotaped GP consultations with related data have been demonstrated in small studies.^10^^,^^11^ Stakeholders believed that collecting such data would be useful for training doctors and quality improvement, but expressed concern about data quality, security, confidentiality, and governance.^10^ Videos provide a more accurate record of consultations including vocal, verbal, and visual behaviour, and a recent review has shown that most people regard video-based research in healthcare settings as acceptable and worthwhile.^12^

**How this fits in**

Understanding of routine consultations between patients and doctors in primary care is limited. High-quality consultation data are challenging to collect, with considerable ethical and practical hurdles. There are no existing archived datasets with permissions in place for reuse. The ‘One in a Million’ database provides a high-quality controlled-access resource for future research and training in UK general practice.

Therefore, the aim of this project was to create a controlled database of high-quality videotaped GP–patient consultations, with linked practice, GP, and patient data, with consent for reuse for the purpose of future research and teaching.

## METHOD

The design was a cross-sectional study of routine GP consultations with adult patients.

### Participant selection and recruitment

A purposive sample of 12 practices from three clinical commissioning groups (CCGs) in the west of England, including urban, suburban, and semi-rural areas of high and low deprivation, was recruited to the study via the NIHR Clinical Research Network. Twenty-three GPs volunteered to have up to 20 routine consultations recorded over two or three half-day sessions between July 2014 and April 2015.

All adults aged ≥18 years seeing study GPs on recording days were handed a study leaflet on arrival, and given the opportunity to discuss the study with a researcher before deciding whether or not to participate and have their consultation recorded (by either video or audio-only). Participating patients were also asked to give consent for the research team and/or other researchers to use data in the future, subject to ethical approval, and for their data to be used for the development of medical and research training materials. Patients could choose to consent to any or all of these activities.

For ethical reasons surrounding ongoing consent, patients were excluded if they were aged <18 years, lacked capacity to give informed consent, were consulting on behalf of a third party, or did not speak English fluently. The sex and ethnic group of patients who declined participation, and the reasons given, were recorded on screening logs.

Patients and doctors completed questionnaires before and after each consultation. The timing and data collected are listed in Table 1.

**Table 1.** Archive data: measures collected, from whom and at which time point

**Data source/when completed**	**Patient**				**GP**	
	**Demographic survey**	**Pre-visit survey**	**Post-visit survey**	**Follow-up survey**	**GP checklist**	**GP survey**
	**Same day as appointment**	**Before appointment**	**Straight after appointment**	**10 days after appointment**	**Straight after appointment**	**First day of data collection**
**Measure**	**Number completed (% of consultations recorded)**					
	***n*= 328 (98.2)^a^**	***n*= 287 (85.9)**	***n*= 301 (90.1)**	***n*= 176 (52.7)**	***n*= 325 (97.3)**	***n*= 23 (100)**
Patient demographics	✓					
Pre-visit expectations^13^		✓				
Control preferences scale^14^		✓				
Perceived influence over consultation outcome^13^		✓				
Health-related quality of life measure^15^		✓	✓	✓		
Perceptions of consultation outcome^13^ and decision making^14^			✓		✓	
Perception of decision making^16^			✓		✓	
Depth of doctor–patient relationship^17^			✓		✓	
Enablement^18^			✓			
Recall of and adherence to treatment recommendations^19^				✓		
Patient beliefs about medicines^20^				✓		
GP clinical certainty of treatment plan^21^ and confidence in patient following treatment plan^21^					✓	
GP demographics						✓
GP work-related burnout,^20^ stress, and job satisfaction^23^						✓

a Percentages calculated from a total number of 334 (with the exception of the GP survey in the final column).

Three months after the index consultation, typed entries relating to the index consultation and any related subsequent entries (reconsulting with the same or another GP or nurse, an out-of-hours visit, or emergency department visit) were extracted from consenting patients’ medical records. Entries were defined as being related to the index consultation if the same symptom or problem was recorded. The number of other, unrelated reconsultations in the same time period was also recorded.

### Consultation data

GPs were asked to videotape (or audiotape according to participant preference) all consultations with consenting adults. All recordings were transcribed verbatim by a professional transcription service, anonymised for names and place names, and the content coded for problems and issues discussed using a published coding tool^24^ based on the *International Classification of Primary Care*, 2nd edition.^25^

### Practice data

Geographical location, urban/rural classification, area-related deprivation score, patient list size, number of GPs at the practice, Quality and Outcomes Framework achievement, and score on doctor–patient communication items from the GP Patient Survey were extracted from practice websites. Consultation rates and number of missed appointments in the month of data collection were extracted from a search applied to practice records systems.

Study data were managed using REDCap electronic data capture tools hosted at the University of Bristol.^26^ Individual-level Index of Multiple Deprivation (IMD) scores were derived from patient postcodes. Descriptive data analyses were undertaken using Stata (version 14). All numerical data were checked for missing values and potential invalid entries.

## RESULTS

### Practice characteristics

Of the 12 participating practices, six were located in areas of high deprivation (IMD 24–69) and six in areas of low deprivation (IMD 0–10). Ten were training practices and list sizes ranged from 6250 to 18 350 patients (mean practice list size 11 999, standard deviation [SD] 4264).

### GP characteristics

Thirteen female and 10 male GPs participated, all of whom were of white ethnic group. Seven were aged <40 years and 16 were aged ≥40 years. The participants had been qualified as GPs for a mean of 18 years (range 2–35 years), and had worked at their current practice for a mean of 10 years and 11 months (range from 6 months to 32 years 1 month).

**Figure 1.**
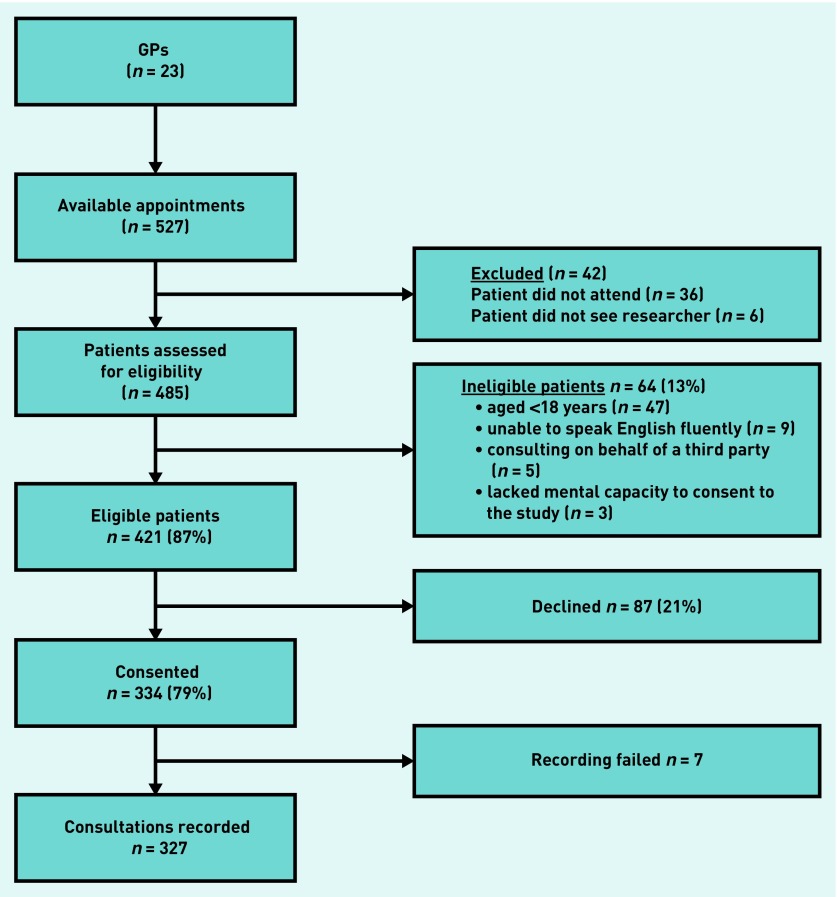
***Flow diagram of patient recruitment and response rates.***

The flow of patients through the study is shown in Figure 1.

### Patient characteristics

The characteristics of the patient sample are shown in Table 2.

**Table 1.** Characteristics of patient sample, *N* = 334

	***n***	**%**
**Sex**		
Female	212	63.5
Male	122	36.5

**Ethnic group**		
White	291	87.1
Other	43	12.9

**Age, years**		
18–34	91	27.2
35–54	94	28.1
55–74	99	29.6
≥75	36	10.8
Not reported	14	4.2

**IMD quintile**		
1st (least deprived)	106	31.7
2nd	54	16.2
3rd	35	10.5
4th	53	15.9
5th (most deprived)	84	25.1
Data unavailable	2	0.6

Three-hundred and twenty-seven (97.9%) patients agreed for their data to be retained for use by the ‘current research team’, 300 (89.8%) for future use by ‘other researchers’, and 299 (89.5%) for ‘the development of medical and research training materials’. Of the recorded consultations, 167 were with patients attending practices in areas of high deprivation (at a consent rate of 77.5%) and 160 at practices in areas of low deprivation (consent rate 81.4%).

### Consultations data

Camera error caused seven out of 334 recordings to fail. Of the 327 successful recordings, 307 (94%) were video and 20 audio-only. The mean duration of consultations was 12 minutes 2 seconds (95% confidence interval [CI] = 11.28 to 12.36; SD 05.14; range 1 minute 19 seconds to 37 minutes 54 seconds). The patient initiated 294 out of 327 consultations (89.9%), with 17 (5.2%) initiated by the doctor or the practice (in the remaining 16 [4.9%] it was not possible to tell). Most of the interactions (275, 84%) were two-party interactions featuring just the patient and a GP, while 52 (16%) featured a third party, typically the patient’s child, partner, parent, and/or carer. Nine were ‘joint’ consultations; double appointments booked for two people to be seen consecutively.

In the 327 recordings, a total of 518 problems were discussed, with an average of 1.58 per consultation (95% CI = 1.49 to 1.68; SD 0.05; median 1; range 1–5). Most problems were raised by patients (*n* = 441; 85.3%), with 66 (12.6%) raised by the GP, and 11 (2.1%) raised by a third party. Table 3 shows the types of problems discussed. Following Procter *et al*,^24^ the dimensions of each problem, that is, the types of ‘issues’ that were discussed with the GP to address the problem, were also classified using the nine issue types shown in Table 4.

**Table 3.** Types of problems discussed (in order of frequency)

**ICPC-2 Code**	**Problem type**	**Frequency**	**%**	**Number of consultations with this problem type**
L	Musculoskeletal	94	18.1	89
P	Psychological	63	12.2	63
D	Digestive	53	10.2	52
R	Respiratory	48	9.3	46
S	Skin	45	8.7	42
K	Cardiovascular	39	7.5	37
A	General and Unspecified	31	6.0	25
U	Urological	23	4.4	23
N	Neurological	22	4.2	21
T	Endocrine/Metabolic and Nutritional	22	4.2	22
W	Pregnancy, Childbearing, Family Planning	21	4.1	21
X	Female Genital	20	3.9	20
H	Ear	11	2.1	11
Y	Male Genital	11	2.1	10
B	Blood, Blood Forming, and Immune Mechanism	9	1.7	9
F	Eye	6	1.2	6
**Total number of problems**			**518**	**100.0**

*ICPC-2 =* International Classification of Primary Care*, 2nd edition.*

**Table 4.** Types of issues discussed

**Issue type/description**	**Frequency**	**%**
Physical		
*Discussion or reference to physical symptoms*	534	31.4

Emotional/psychological		
*Psychological or emotional dimensions or consequences of the problem*	97	5.7

Social		
*The consequences of the problem on the patient’s normal social roles or activities*	58	3.4

Administrative		
*Requests for letters and sick notes; making referrals for further consultations; making repeat appointments*	74	4.4

Medication related		
*Relating to any existing medication, prescription, or administration of new medication*	402	23.7

Order/refer for tests		
*Raising or resolving the need for tests or investigations to be done beyond the current consultation*	117	6.9

Discuss test results		
*Issues that follow up test results, investigations, or treatments (other than medication) that were performed before the consultation*	114	6.7

Behavioural health prevention		
*Information given or sought relating to patient-actioned prevention, self-management, or risk management behaviours*	216	12.7

Medicalised health prevention		
*Information given or sought relating to GP-actioned patient prevention, self-management, or risk management issues*	125	7.4

### Survey data

The pre-consultation survey was completed by 287 patients, and 301 filled in the post-consultation survey on the day of data collection, or very soon afterwards. A follow-up survey, which was sent either by post or e-mail 10 days after the recorded consultation, was completed by 176 patients, representing a return rate of 52.7%, with rates particularly low in the more deprived neighbourhoods. All 23 GPs completed the GP surveys and 325 post-consultation checklists were completed. Details of the numbers of completed surveys are included in Table 1.

### Medical records data

Medical record entries were collected for the index consultations of 311 patients. Of these, 227 out of 311 (73.0%) patients had reconsulted at least once. For reconsultations, 128 of these patients had reconsulted with the same GP and 143 with another GP in the practice for the same problem, with some patients seeing both the same doctor and a different doctor.

## DISCUSSION

### Summary

An archive of recordings was successfully created of over 300 routine general practice consultations with linked practice and GP data, plus pre- and post-visit data from patients and medical records, with permissions in place for reuse by other *bona fide* researchers. The archive is stored digitally in the University of Bristol Research Data Repository, where a more detailed description of the contents may be found. As controlled data, reuse in future studies is governed by approval by an NHS ethics committee and the University of Bristol Data Access Committee. The recorded consultations have been transcribed verbatim, anonymised for spoken names and place names, and the problems and issues discussed within each consultation have been coded. An electronic database has been created that allows the data to be searched according to numerous variables at practice, GP, patient, or visit-level, with all data points linked to the index recordings.

### Strengths and limitations

The data archive created in this study appears to be the only one of its kind in the UK, allowing access for further research. It contains qualitative and quantitative data including descriptive data for each practice setting, high-quality videotapings, transcripts, and longitudinal questionnaire and medical records data. By prospectively collecting data, the precursors and immediate and more distal outcomes of consultations can be examined. Data were collected equally across areas of low deprivation and areas of high deprivation. Limitations include practices and GPs self-selecting to take part in the study, and the proportion of training practices being substantially higher than across general practice as a whole. By selecting practices within areas of high deprivation and areas of low deprivation, a wide range of patient characteristics have been included but at the expense of generalisability to practices with average deprivation. The recordings are also limited in that they provide a snapshot of 1 to 2 days of consulting for a small number of GPs, clustered within a smaller number of practices. Telephone consultations are increasingly common and these were not captured. All the practices were located across three CCGs in the west of England and consultation behaviour may be different in other areas. For all these reasons, the generalisability of the sample, and the potential to detect differences between subgroups, are limited. The aim of the study has been achieved, however, with standard operating procedures and tools developed to enable future collection of similar data from other areas, which can be added to the archive over time.

### Comparison with existing literature

This study has taken forward the work of Rushmer *et al*^10^ and Williams *et al*,^11^ demonstrating that GPs and patients are willing to have their consultations recorded and linked data collected and archived for controlled future use. The high rates of consent to videotaping are similar to those reported by Salisbury *et al*.^27^ This study also builds on the legacy of Byrne and Long’s^5^ UK study of GP consulting behaviours to provide the foundations for a database that can enable not one but many future studies of primary care in situ.

### Implications for research and practice

The recordings held in the database will support a wide range of future qualitative, quantitative, and mixed-methods research. This could include studies of healthcare communication including comparative studies and those assessing the relationship between consultation processes and outcomes; proof of concept studies such as the development of new communication interventions; and methodological studies such as the development of new coding tools. Furthermore, the recordings may also be of interest to others beyond medical research, such as linguists. Data sharing provides considerable added value in terms of minimising data collection costs, reduced environmental impact, and patient and practice burden. This will support low-cost studies including doctoral-level research, thus building research capacity in primary care. The archive can also be used for development of medical and research methods teaching/training materials with high face validity.

Having developed the necessary study materials, obtained relevant ethical approvals, and demonstrated the feasibility of collecting videotapings of routine GP consultations for the purpose of creating an archive for future research/teaching, the present authors welcome additional consultation data from other researchers to help grow the database. It is hoped that, as well as more consultations, future deposits using the current study protocols will include other aspects of general practice (for example, telephone consultations and home visits) and other practitioners (for example, nurse consultations).

Patient–doctor consultations are at the core of primary care. Greenhalgh *et al* argued that the drive for evidence-based medicine has overlooked the everyday context of clinical practice.^28^ Each recorded consultation in the present database represents a snapshot of the context of clinical practice for one individual in the million or so consultations that take place in England alone each day. Greater understanding of the content and conduct of these consultations, through research and teaching using the archive, will help improve the process and outcomes in primary care.
